# Assessment of influenza virus and coronavirus tropism, replication competence and disease severity in ex vivo and in vitro cultures of the human respiratory tract

**DOI:** 10.1099/jgv.0.002281

**Published:** 2026-07-03

**Authors:** Denise I.T. Kuok, Angel P.Y. Ma, Rachel H.H. Ching, Ka Chun Ng, Jae W. Lee, Michael A. Matthay, Yi Guan, John M. Nicholls, Leo L.M. Poon, J.S. Malik Peiris, Kenrie P.Y. Hui, Michael C.W. Chan

**Affiliations:** 1 School of Public Health, LKS Faculty of Medicine, The University of Hong Kong, Hong Kong, PR China; 2 Centre for Immunology and Infection (C2i), Hong Kong Science Park, Hong Kong, PR China; 3 Department of Anesthesiology, University of California, Los Angeles, USA; 4 Department of Medicine and Anesthesiology, University of California San Francisco, San Francisco, USA; 5 Department of Pathology, School of Clinical Medicine, LKS Faculty of Medicine, Queen Mary Hospital, The University of Hong Kong, Hong Kong, PR China

**Keywords:** airway epithelium, epithelial cells, influenza, lung injury, SARS, viral infection

## Abstract

The emergence of animal influenza viruses circulating in poultry and human populations poses a significant public health threat, yet current risk assessment tools that connect surveillance data to human transmission risk and disease severity are lacking. To address this, we employed a semi-quantitative approach to analyze virus tropism and replication competence, conducting risk assessments of influenza and coronavirus adaptation to human transmission in an *ex vivo* model, and evaluating virus-induced impairment of alveolar fluid clearance (AFC) *in vitro* as a correlation of disease severity. Our results showed that seasonal influenza A H1N1, H3N2, influenza B, MERS-CoV, and SARS-CoV exhibited productive viral replication and tissue infection in bronchial tissues, whereas wild bird surveillance isolates such as H5N3 and H7N1 showed minimal replication when compared to pandemic H1N1 and highly pathogenic avian influenza (HPAI) H5N1. Notably, differential lung viral replication and tissue tropism were detected for H5N6 and H9N2. HPAI H5N1, H7N9, MERS-CoV, and SARS-CoV caused more severe AFC impairment than seasonal H1N1, H3N2, and influenza B viruses, correlating with their clinical severity. Overall, these findings revealed an important association between viral tropism and human transmissibility in *ex vivo* explants, as well as the impairment of AFC *in vitro*, which aligns with the clinical manifestations of disease severity across different viral strains.

**Impact Statement**

This study integrates a semi-quantitative assessment of viral tropism and replication competence with functional evaluation of alveolar fluid clearance, providing a novel framework for correlating viral infectivity with disease severity. It fills a critical gap by linking *ex vivo* viral behaviour with potential clinical outcomes, thereby enhancing our understanding of factors influencing viral transmissibility and pathogenicity.

The research methodology is broadly applicable to viral surveillance, risk assessment and understanding respiratory disease pathogenesis, benefiting virologists, epidemiologists, clinicians and public health officials.

The findings represent a significant step forward by connecting viral tissue tropism, impairment of alveolar function and clinical disease severity. The study also provides an important mechanistic basis that enhances existing risk assessment tools, moving beyond descriptive surveillance data towards functional and pathogenic insights. This offers a more nuanced understanding of how specific viral characteristics translate into human disease risk, thereby informing more targeted surveillance and intervention strategies.

## Introduction

Pandemics arise at unpredictable intervals and spread worldwide within weeks of high infection attack rates, potentially associated with significant morbidity and mortality [1]. Vaccines will not be available in time to mitigate the first wave of pandemic. Therefore, active surveillance programmes have been carried out worldwide to identify and risk assess animal viruses circulating in wild birds, poultry, swine and other animals, so that seed viruses for candidate vaccine and sometimes actual vaccines can be prepared preemptively. The 2009 pandemic virus emerged through the reassortment of swine North American triple reassortant H1N1/H1N2 viruses (which had caused zoonotic diseases in North America) and swine viruses of the Eurasian-avian lineage [2]. The failure to identify these swine North American viruses as potential pandemic threats was not a fail of surveillance but rather a gap in the risk assessment. Since then, algorithms have been developed for systematic risk assessment of animal influenza viruses for pandemic threat to facilitate the selection of animal viruses as vaccine candidates. CDC Influenza Risk Assessment Tool (IRAT) and WHO Tool for Influenza Pandemic Risk Assessment (TIPRA) are used to evaluate viruses for pandemic threat in relation to emergence risk and public health impact or disease severity [3,4]. These assessments are based on virus properties, population attributes and virus ecology. Individual parameters that feed into such algorithms need further refinement.

One of the key parameters used to assess the risk of an animal virus becoming transmissible between humans is the ability of virus to infect and replicate in human upper respiratory tract. Currently, this is indirectly assessed by investigating the binding of virus to sialic acids that are α2–6-linked to galactose, which is believed to be the type of receptors found in the human upper airways [5]. However, there is a wide diversity of mono-antennary, bi-antennary or poly-antennary glycans, and it is still unclear which of these are present in the human upper airways [6]. Some of the glycans present in the human upper airways are not available in synthetic form and not found in glycan arrays currently used in assessing receptor binding of influenza viruses. We have therefore developed an alternate and more direct strategy of using *ex vivo* cultures of human upper airways to investigate tropism and replication competence of influenza viruses [7,9].

Virus binding to bronchus tissues [10] or infection of *ex vivo* human bronchus [7,9] is highly correlated with viruses manifesting airborne transmission in ferrets – another reliable correlate of transmissibility in humans. Given the possibility of donor variation, it has been difficult to compare viral replication competence in separate experiments of virus-infected *ex vivo* cultures in a reproducible manner. Here, we propose to quantitatively analyse the viral replication competency of a panel of influenza viruses in the human respiratory tract. This analysis will compare these viruses to two reference virus strains: the 2009 H1N1 pandemic virus, which transmits efficiently in humans, and the highly pathogenic avian influenza (HPAI) H5N1 virus, which has not been able to acquire human transmissibility.

*Ex vivo* culture of lungs does not necessarily provide a means of assessing severity of animal influenza. While both HPAI H5N1 viruses and seasonal influenza viruses readily infect human lungs [8,10], HPAI H5N1 virus causes more severe lung disease than seasonal influenza viruses [11,12]. A major cause of severe disease and deaths in HPAI H5N1-infected patients is acute respiratory distress syndrome, a severe form of acute lung injury (ALI) where pulmonary oedema fluid accumulation results in impaired alveolar fluid clearance (AFC) [13]. We have previously shown differences between HPAI H5N1, low-pathogenic avian influenza (LPAI) H7N9 and seasonal H1N1 viruses in their impact on AFC in an *in vitro* lung injury model of primary alveolar epithelial cells (AECs) [14]. In these studies, we also concluded that the effect of these viruses on AFC was driven by soluble mediators released into the cell culture supernatant and not by virus-induced cytopathic effect. We thereby defined this experimental model as a correlation of disease severity of avian, human influenza viruses and coronaviruses. In summary, we advanced the risk assessment of human transmissibility and disease severity of influenza viruses as to prepare for future pandemics.

## Methods

### Influenza viruses and coronaviruses

Influenza viruses and coronaviruses used in this study and their virus isolation origin were listed in Table S1 (available in the online Supplementary Material). Wild bird faecal samples were collected during routine surveillance at the Hong Kong Mai Po Natural Reservoir. All influenza viruses were passaged in Madin-Darby Canine Kidney, whereas coronaviruses in Vero E6 or MRC-5 cells. Viral titres were determined by median TCID_50_ as described [8] (see Supplementary Material). All experiments were performed inside a biosafety level-3 facility.

### Infection of *ex vivo* cultures of human bronchus and lung

Fresh and non-malignant lung and bronchus tissues were taken from patients who underwent lung resection. Patient consents were obtained, and the study was approved by the Institutional Review Board of the University of Hong Kong and Hospital Authority (approval no: UW 14–119). Tissue maintenance, infection protocol and analysis were as described previously [7,9,15] (see Supplementary Material). Optimal fixation times were selected for different viruses for the assessment of cell tropism. Infection of ciliated and non-ciliated epithelial cells was assessed by a clinical pathologist. Three sections were analysed for immunohistochemistry (IHC), with a representative image selected for comparison.

### Area under the curve analysis of viral replication competence in *ex vivo* culture

Viral replication competence in *ex vivo* cultures of human bronchus and lung was presented as area under the curve (AUC) by GraphPad Prism v5.0 (GraphPad Software, USA). AUC was determined between virus replication kinetic curves and the detection limit of TCID_50_ assay (10^1.5^) at 24 and 48 hours post-infection (hpi) [16] using Prism. For *ex vivo* bronchus culture, AUC of reference strain pandemic H1N1 (A/Hong Kong/415742/2009) was set as 100 and HPAI H5N1 (A/Hong Kong/483/1997) as 0 for normalization of each test virus (see Supplementary Material).

### *In vitro* lung injury model

Primary human AECs on apical Transwell inserts with a 0.4 µm pore size (Corning) were infected with influenza viruses and coronaviruses at MOI of 0.1 and 1 as previously described [14] (see Supplementary Material). Based on preliminary optimization experiments, different MOI values were used for influenza viruses and coronaviruses to ensure sufficient viral replication for comparative analysis. After infection, cells were replenished with growth medium (SAGM™, Lonza, USA) containing 12.5 µg 70 kDa FITC-labelled dextran (Sigma). Mock-infected AECs were used as negative control. Net AFC was determined by change in fluorescent intensity of dextran over a 24 h infection period [14] and normalized to reference strains to define relative AFC (see Supplementary Material). Viral titres of culture supernatant of AECs were measured at 1, 24 and 48 hpi by TCID_50_ assay and AUC was calculated for 24–48 hpi using Prism. To test the impact of MOI on AFC, AECs were infected with H1N1, H3N2 and H1N1pdm of MOI 0.1, 1 and 10 and HPAI H5N1 at MOI 0.1. AFC was measured at 16 hpi to minimize direct cytopathic effect on AFC since cell death was observed with MOI 10 at 24 hpi.

### Statistical analyses

*Ex vivo* infections and AFC of multiple viruses were compared using one-way ANOVA with Bonferroni post-tests in Prism. Spearman’s rank correlation coefficient between viral replication (AUC) on AECs and AFC of all viruses was calculated using Prism.

## Results

### Replication competence and tissue tropism of influenza viruses and coronaviruses in human bronchus

**Fig. 1.**
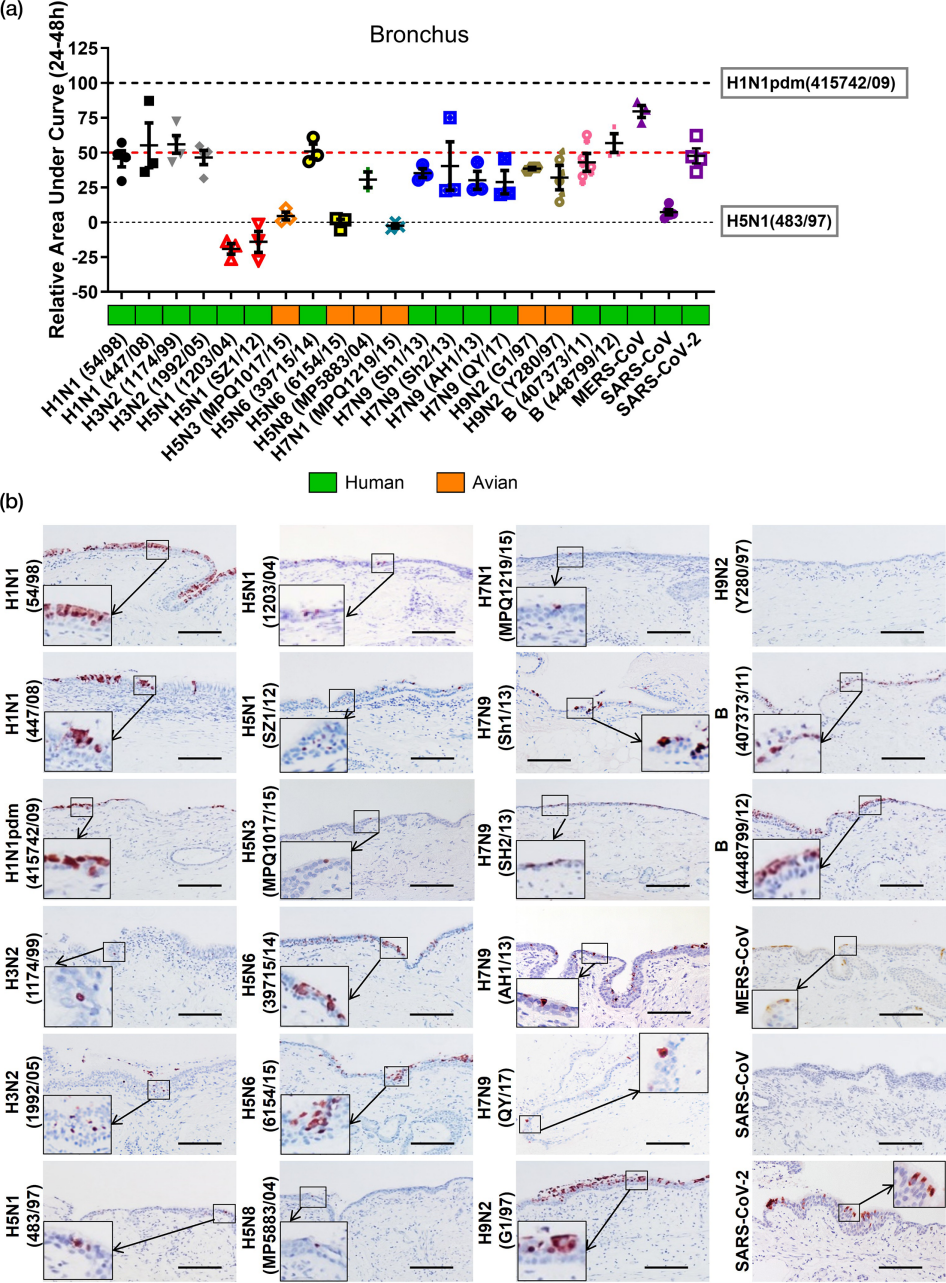
Tissue tropism and replication competency of influenza viruses and coronaviruses in *ex vivo* cultures of human bronchus. *Ex vivo* human bronchus tissues were infected with 10^6^ TCID_50_ ml^−1^ infectious dose of different influenza subtypes and coronaviruses at 37 °C, tissues were formalin-fixed and paraffin-embedded at 24 hpi (for influenza A) and 48 hpi (for influenza B and coronaviruses). (**a**) Viral titres in the culture supernatant of infected tissues were measured at 1, 24 and 48 hpi using TCID_50_ assay. AUC was calculated from replication kinetic between 24 and 48 hpi (*n*≥3). Relative AUC was calculated by normalizing to H1N1pdm (415742/09) set as 100 and HPAI H5N1 (483/97) set as 0 and presented as dot plot with mean±sem. Red dotted line represents 50% relative AUC. (**b**) Sections were immunohistochemically stained for nucleoprotein with specific antibodies against respective viral nucleoprotein (shown in reddish brown). Magnification is ×200 and scale bar 100 µm. ≥61% APC of total epithelial cells are considered as high infection, 31–60% APC as moderate and 1–30% APC as minimal infection.

Experimental workflow (Fig. 1): To assess viral replication and tissue tropism, *ex vivo* human bronchus tissues were infected with 10^6^ TCID_50_ ml^−1^ of various influenza viruses and coronaviruses, then incubated at 37 °C for 24 h for influenza A and 48 h for influenza B and coronaviruses. Post-infection, tissues were fixed for histological analysis, and supernatants were collected to measure viral titres via TCID_50_ assay. The AUC for viral replication between 24 and 48 h was calculated and normalized against the pandemic H1N1 virus (set as 100) and the HPAI H5N1 virus (set as 0). Tissue sections underwent immunohistochemical staining for viral nucleoproteins to determine the percentage of antigen-positive cells (APCs) among total epithelial cells, categorizing infection levels as high (≥61%), moderate (31–60%) or minimal (1–30%). This enabled correlation of replication kinetics with tissue infectivity and cellular tropism.

Seasonal influenza A, B and MERS-CoV had substantial replication on *ex vivo* human bronchus cultures with relative mean AUC ≥41.1 (minimal AUC index). AUC is defined by the area between TCID_50_ detection limit and the replication kinetic curve at 24 and 48 hpi. Since the 2009 H1N1 pandemic is the most recently emerged pandemic virus that has shown sustained human-to-human transmission and HPAI H5N1 viruses have not become transmissible in humans despite repeated zoonotic infections for over two decades, these two viruses were used as the ‘positive’ and ‘negative’ control reference strains for AUC normalization of different experiments [8,10,17]. AUC of pandemic H1N1 (415742/09) with high human bronchus infection was set as 100 and HPAI H5N1 (483/97) with minimal human bronchus infection was set as 0 for AUC normalization. Seasonal human influenza H1N1 (54/98, 447/08), H3N2 (1174/99, 1992/05), H5N6 (39715/14), influenza B viruses (407373/11, 448799/12) and SARS-CoV-2 had ~50% relative AUC with the lowest AUC index of 42.97 (Figs 1a and S1A, C). In contrast, HPAI H5N1 (1203/04, SZ1/12) and LPAI surveillance isolates H5N3 (MPQ1017/15), H7N1 (MPQ1219/15), H5N6 (6154/15) and SARS-CoV had relative AUC comparable to the control HPAI H5N1 (483/97) virus. Human isolate HPAI H5N6 (39715/14) had markedly higher bronchus replication than avian HPAI H5N6 (6154/15) virus (similar to our previous study [18]). Interestingly, H7N9 (Sh1/13, Sh2/13, AH1/13, QY/17) and H9N2 (G1/97, Y280/97) had ~40% relative AUC comparable with some seasonal influenza viruses. MERS-CoV replicated better in human bronchus than SARS-CoV, as shown by the 75% relative AUC of MERS-CoV (EMC).

The pattern of viral APCs (including ciliated and non-ciliated epithelial cells) of human bronchus in the IHC staining suggested different levels of infectivity and cellular tropism (Fig. 1a, b and Table S2). Extensive bronchus infection was observed with seasonal H1N1, H1N1 pandemic, H7N9 (Sh2/13), H9N2 (G1/97) and influenza B viruses (≥61% APC) of total DAPI-positive cells); moderate infection with H5N6, H7N9 (Sh1/13),MERS-CoV and SARS-CoV-2(31–60%APC), and minimal infection with HPAI H5N1, H5N3, H5N8, H7N1, H7N9 (AH1/13, QY/17), H9N2 (Y280/97) and SARS-CoV (1–30% APC). Interestingly, H3N2 (1174/99, 1992/05) viruses had low numbers of infected bronchus epithelial cells, although they had moderate viral replication (Fig. 1a, b). Generally, there are no notable differences in infectivity by morphological analysis between ciliated and non-ciliated epithelial cells among different influenza subtypes, as observed with IHC staining (Fig. 1b).

### Tissue tropism and replication competence of influenza viruses and coronaviruses in human lung

**Fig. 2.**
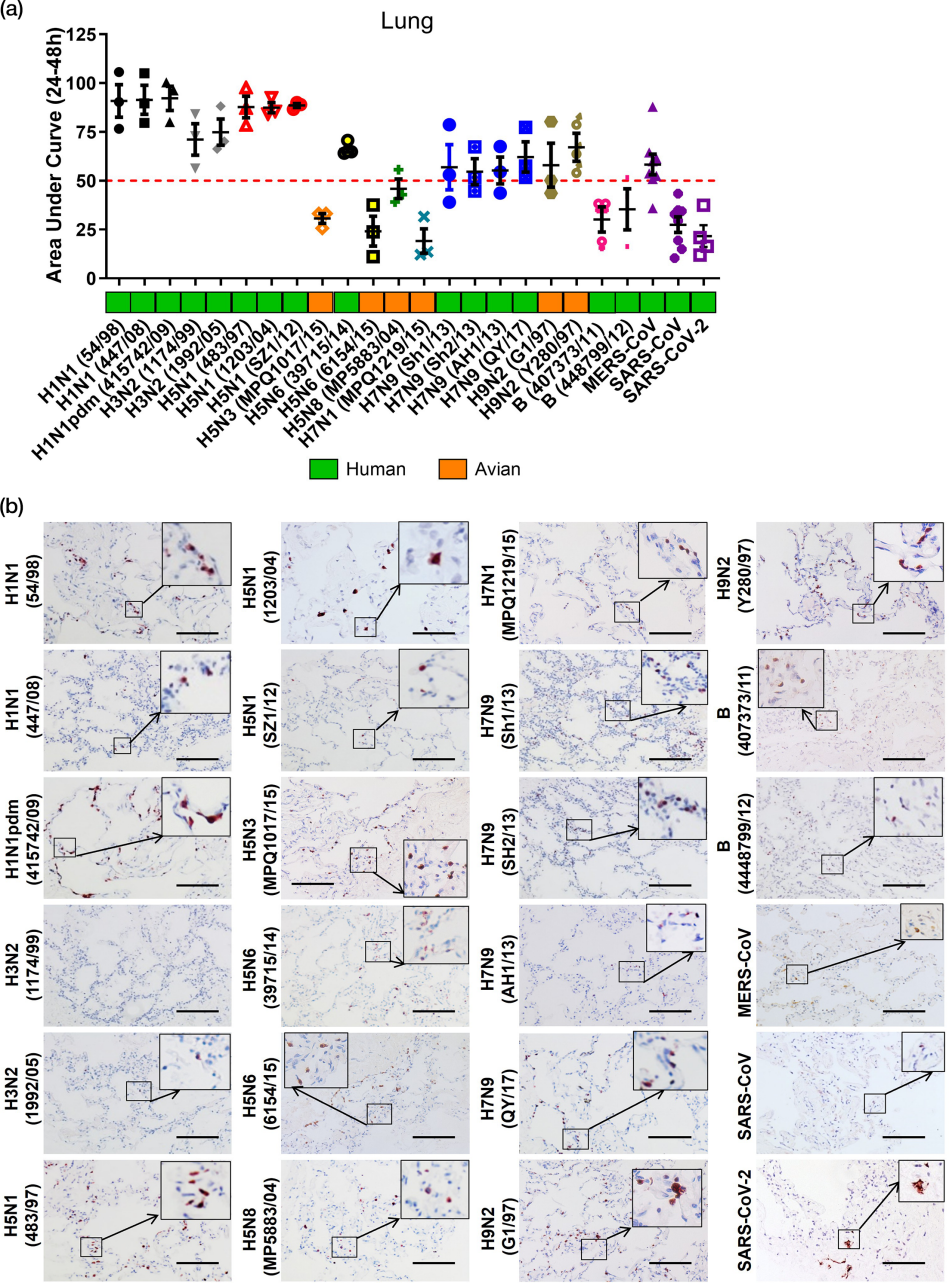
Tissue tropism and replication competence of influenza viruses and coronaviruses in *ex vivo* infected human lung tissues. *Ex vivo* human lung tissues were infected with 10^6^ TCID_50_ ml^−1^ infectious dose of different influenza subtypes and coronaviruses at 37 °C, tissues were formalin-fixed and paraffin-embedded at 24 hpi (for influenza A) and 48 hpi (for influenza B and coronaviruses). (a) Viral titres in the culture supernatant of infected tissues were measured at 1, 24 and 48 hpi using TCID_50_ assay and plotted as AUC in dot plot with mean±sem (n ≥ 3). Red-dotted line represents 50% relative AUC. (b) Sections were immunohistochemically stained for nucleoprotein with specific antibodies against respective viral nucleoprotein (shown in reddish brown). Magnification is ×200 and scale bar 100 µm. ≥61% APC of total epithelial cells are considered as high infection, 31–60% APC as moderate and 1–30% APC as minimal infection.

Experiment workflow (Fig. 2): *Ex vivo* human lung tissues were infected with various influenza viruses and coronaviruses at 10^6^ TCID_50_ ml^−1^ and incubated at 37 °C, with tissues fixed at 24 h for influenza A and 48 h for influenza B and coronaviruses. Viral titres in the supernatants were measured at 1, 24 and 48 hpi, and the AUC between 24 and 48 h was calculated to assess replication efficiency. Immunohistochemical staining for viral nucleoproteins revealed that nearly all infected lung tissues contained APCs.

Most of the viruses replicated efficiently between 24 and 48 hpi in human lung tissues except H5N3 (MPQ1017/15), H5N6 (6154/15), H7N1 (MPQ1219/15), influenza B viruses, SARS-CoV and SARS-CoV-2 (about half or less replication competence of other viruses) (Figs 2a and S1B). Viral APC are found in almost all infected lung tissues except H3N2 (1174/99) (Fig. 2b), even though this virus had evidence of viral replication (Fig. 2a). Seasonal H1N1, H1N1 pandemic, HPAI H5N1, H5N8, H7N9 and H9N2 had extensive positive staining of alveolar epithelium (≥61%APC); H5N3, H5N6, influenza B and MERS-CoV had moderate positive staining (31–60% APC); and H3N2 (1992/05), H7N1, SARS-CoV and SARS-CoV-2 (1–30% APC) had least positive staining. Both H1N1 pandemic and HPAI H5N1 replicated efficiently in human lungs despite manifesting different disease severity [19,20]. Thus, it is apparent that virus replication in the alveolar epithelium is not the key determinant of disease severity.

### Evaluation of alveolar fluid clearance as a correlate of disease severity

**Fig. 3.**
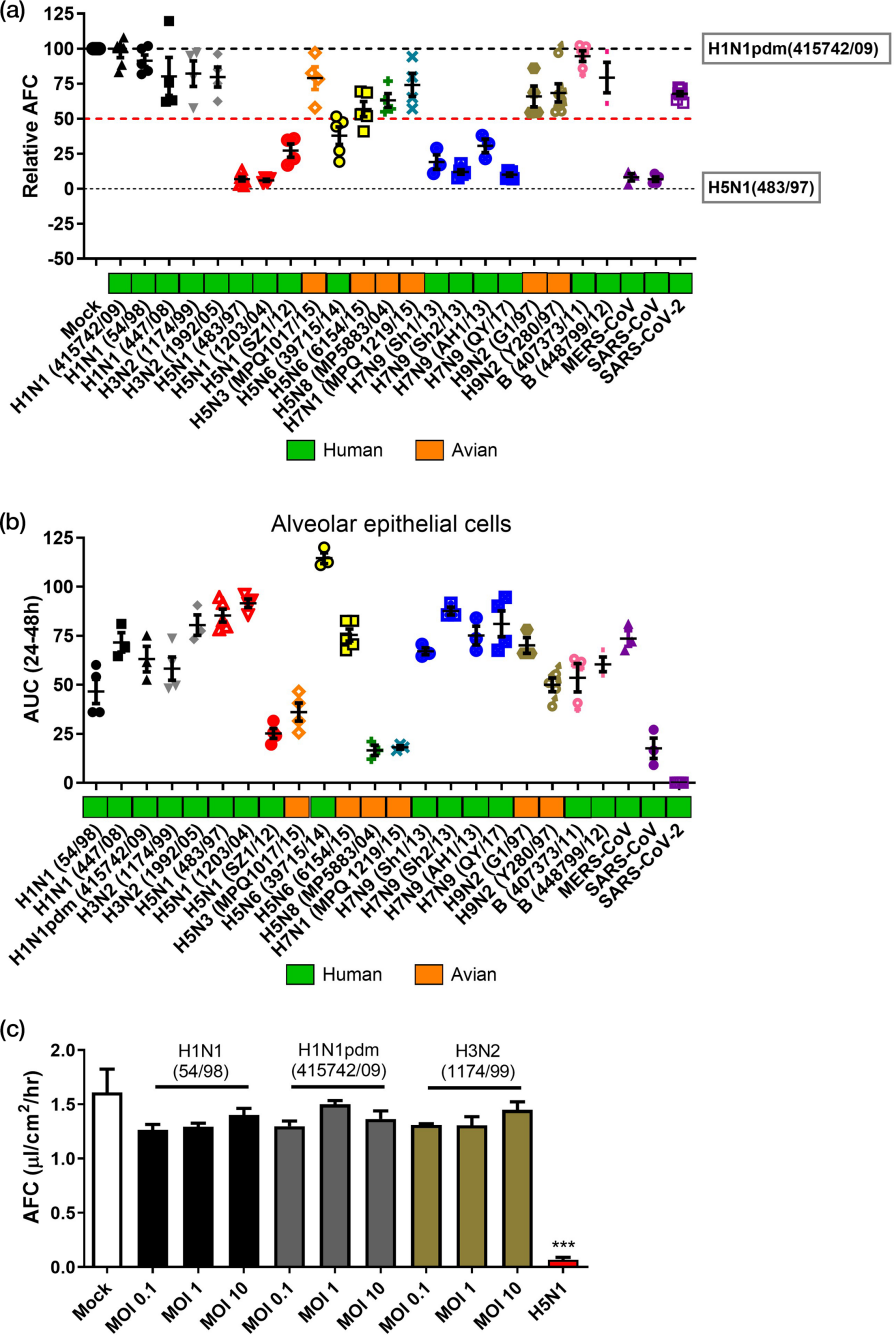
Different levels of AFC induced by influenza viruses and coronaviruses infection as indicator of disease severity in the *in vitro* lung injury model. (a) AECs on apical Transwell were infected by different influenza viruses at MOI 0.1 and coronavirus at MOI 1. Fluorescent reading of AECs was measured at 5 min and 24 hpi in the culture supernatant, and AFC was calculated in μl cm^–2^ hr^–1^. Relative AFC was calculated by normalizing to H1N1pdm (415742/09) set as 100 and HPAI H5N1 (483/97) set as 0 and presented in dot plot with mean±sem (n ≥ 3). Red-dotted line represents 50% relative AFC. (b) Viral titre in the culture supernatant of infected AECs was measured at 1, 24 and 48 hpi using TCID_50_ assay. AUC was calculated from replication kinetic between 24 and 48 hpi (n ≥ 3) and presented as dot plot with mean±sem. (c) AECs in transwell were infected by influenza H1N1 (A/HK/54/98), pandemic H1N1 (415742/09) and H3N2 (1174/99) viruses at MOI 0.1, 1 and 10 and HPAI H5N1 (483/97) at MOI 0.1. Fluorescent reading was measured at 5 min and 16 hpi, and AFC was calculated and presented as dot plot (n ≥ 3, mean±sem).

Experimental workflow (Fig. 3): We compared AFC impairment caused by different influenza and coronavirus infections using an *in vitro* lung injury model. HPAI H5N1 (483/97) and H1N1 pandemic (415742/09) viruses served as reference strains, representing severe and mild disease, respectively; AFC was normalized with H5N1 set as 0 and H1N1 pandemic as 100.

Previously, we demonstrated using the *in vitro* lung injury model that HPAI H5N1 (483/97) virus-infected AECs had impaired AFC, whereas seasonal H1N1 (54/98) virus had minimal impact on AFC [14,21]. Clinically, HPAI H5N1 often causes severe respiratory diseases, whereas H1N1 pandemic generally causes milder diseases, although more widely transmitted in humans [19,20]. Given the distinct disease severity associated with HPAI H5N1 (483/97) and H1N1 pandemic (415742/09) viruses, we used these as the two reference strains to compare level of AFC impairment. Similar to bronchus replication kinetics, net AFC for HPAI H5N1 was set as 0 and H1N1 pandemic as 100. AFC of virus infected-AECs was measured at 24 hpi (Fig. S2A, B) and normalized to these two viruses. Seasonal H1N1, H3N2, H5N3 and influenza B viruses had similar AFC as the H1N1 pandemic virus (>75% relative AFC) (Figs 3a and S2C). In contrast, H5N8, H7N1, H9N2, H5N6 and SARS-CoV-2 viruses had relative AFC scores of ~50–75%, whereas H5N1 (1203/04), H7N9, MERS-CoV and SARS-CoV had low relative AFC scores of ≤25%, similar to HPAI H5N1 (483/97). These HPAI H5N1 and H7N9 viruses are known to cause more severe lung disease than the other influenza viruses [22].

All the viruses replicated at least 1 log above the detection limit (0.15 AUC or 10^1.5^ TCID_50_ ml^−1^) (Figs 3b and S2D). H5N1 (SZ1/12), H5N3, H5N8, H7N1, SARS-CoV and SARS-CoV-2 had relatively low replication, half or less than the level of replication of other viruses. To determine the impact of MOI on AFC in the *in vitro* model, AECs were infected with seasonal H1N1 (54/98 and 1174/99) and pandemic H1N1 (415742/09) viruses at increasing MOI from 0.1 to 10. AFC was not changed with increasing viral titres at 16 hpi when compared to that of H5N1 (483/97) virus at MOI 0.1 (Fig. 3c). Thus, AFC was not affected by different MOI. A moderately negative correlation (Spearman r=−0.6439, *P*=0.0029, **P<0.01) between viral replication (AUC 24–48 h) and AFC of influenza viruses was calculated from this study suggesting efficient replication may not necessarily impair fluid transport to some extent (Fig. S3).

## Discussion

Surveillance and risk assessment of animal influenza viruses for pandemic threat is an important aspect of pandemic preparedness. Current risk assessment algorithms IRAT and TIPRA depend on assessment of a candidate virus in a number of hosts and ecological characteristics to define the risks of an animal influenza virus to transmit efficiently between humans and the severity of a pandemic if such virus is to acquire human transmission. One key parameter with heavy weighting for assessing the risk of a virus acquiring transmissibility in humans is the potential to infect and replicate in the human upper airways. Currently, this is largely assessed indirectly by virus binding to receptors found on the epithelial cells of the upper airways. We had previously shown a more direct approach of using *ex vivo* cultures of human airways to investigate whether a virus can infect and replicate in these epithelial cells. However, previous methods lacked reproducibility and quantitation. With our well-characterized *ex vivo* models (Fig. S4) and by using two reference viruses representing a virus with known pandemic potential (i.e. the 2009 pandemic H1N1 virus) and one that has been zoonotic for over two decades without acquiring transmissibility between humans (i.e. HPAI H5N1) as two polar ends of the spectrum for bronchus replication and AFC analyses, we are now able to provide a semi-quantitative and normalized approach to supplement data from the *ex vivo* cultures of human bronchus for pandemic risk assessment of animal influenza viruses.

We used this strategy to assess seasonal H1N1 and H3N2 viruses that are known to transmit efficiently between humans, zoonotic HPAI H5N1, HPAI H5N6, H7N9 (all LPAI except HPAI (QY/17)), HPAI H5N8 and other LPAI viruses (including the surveillance isolates) that have not been reported to cause zoonotic disease [23,24]. The relative AUC of bronchus replication for each virus is in broad agreement with current consensus on the transmission potential of these viruses, derived from studies on receptor binding, airborne transmission in experimental ferret models and clinical observations. HPAI H5N1 (1203/04, SZ1/12) viruses with the H5 HA derived from the A/goose/Guangdong/1/1996-lineage have comparable low viral replication in bronchus to the reference HPAI H5N1 A/Hong Kong/483/1997 virus. H5N1 viruses do not transmit by airborne route in ferrets, do not bind to alpha-2,6-linked glycans that are found on the human upper airways and have not demonstrated sustained transmission in humans although there have been over 800 zoonotic infections since they were first detected in Hong Kong in 1997 [25]. HPAI H5N6 (39715/14, 6154/15) and H5N8 (MP5883/04) viruses also have HA derived from A/goose/Guangdong/1/96-lineage but are from the clade 2.3.4.4 [18,24]. While H5N6 viruses have caused zoonotic disease, H5N8 viruses have not. Interestingly, H5N6 (39715/14) virus isolated from a human zoonotic infection showed similar productive bronchus replication as seasonal influenza viruses (Fig. 1a). H5N6 viruses have been reported to have dual binding to both alpha-2,6-linked and alpha-2,3-linked glycans found in the upper human airways and bind to human upper airway epithelium [18,26], and this may explain why one of our H5N6 viruses had relatively high replication competence in the human *ex vivo* bronchus cultures. However, these viruses did not manifest airborne transmission in experimentally infected ferrets [26,27]. Two LPAI H5N3 and H7N1 viruses isolated from wild aquatic birds, which have not shown any zoonotic infection, had low bronchus replication like the HPAI H5N1 viruses. The observed discrepancy between influenza B and H3N2 viruses, where influenza B shows moderate replication with high infected cell counts, but H3N2 displays similar replication levels with fewer infected cells, may be due to differences in replication kinetics. The delayed replication initiation for influenza B and H3N2, possibly occurring after 24 hpi, could result in comparable AUCs but differing IHC signals depending on fixation timing. Incorporating TCID_50_ kinetic curves in future studies would provide a clearer understanding of the replication dynamics of the different viruses.

Four H7N9 viruses isolated from humans between 2013and2017 demonstrated moderate bronchus replication (AUC range 27.8–43.5). This is concordant with data that suggest these viruses to manifest some, albeit inefficient, airborne transmission in experimentally infected ferrets, and reportedly have dual binding to both alpha-2,3-linked and alpha-2,6-linked glycans [23,28] and bound to epithelium of human upper respiratory tract [29]. Furthermore, they appeared to have much higher zoonotic potential, with 1,568 confirmed cases being reported to the WHO since their first detection in 2013 [30]. H9N2 viruses with this similar bronchus replication also displayed dual receptor binding to both alpha-2,3-linked and alpha 2–6 receptors [31] but had no evidence of airborne transmission in ferrets [32]. Although MERS-CoV and SARS-CoV had different human bronchus replication, they both have found apparent zoonotic transmission and limited human-to-human transmission [33].

Assessing the potential severity of an animal virus if it has acquired transmissibility in humans is an important second dimension of pandemic risk assessment. This is particularly important for individuals or groups with heightened disease severity, such as in the elderly and in those with underlying diseases [34]. Currently, disease severity is assessed primarily from severity of human zoonotic disease. The pitfall is that only more severe zoonotic infections are likely to be recognized, thus skewing case reports towards greater severity. It is also recognized that no animal model is a good surrogate for human diseases. Since ALI is a pathogenic result of severe influenza infection, we exercised our *in vitro* ALI model to assess the impact of each virus on AFC. It is also the first study to explore the ability of coronaviruses to affect fluid transport *in vitro*. Interestingly, there was a moderately negative correlation between viral replication in AECs and relative AFC (Fig. S3). HPAI H5N1 viruses have high viral replication and low relative AFC which gave rise to a negative correlation. However, pandemic H1N1 with moderate viral replication and high relative AFC has little or moderate correlation. Furthermore, certain viruses, such as H5N1 (SZ1/12) and SARS-CoV, demonstrate a contrasting phenotype. These viruses exhibit relatively low levels of replication but are associated with pronounced AFC impairment. This supports the statement that ALI is not determined by virus tropism on the lung alveolar epithelium alone, but is more related to the nature of mediators released from these infected cells, viral tropism, induction of inflammatory responses, or direct cytopathic effects. The pronounced AFC impairment observed with H5N1 and SARS-CoV despite limited replication underscores the importance of exploring additional mechanisms, including immune-mediated damage or viral proteins that interfere with epithelial ion transport and barrier integrity. Our previous studies demonstrated that the effect of an influenza virus on AFC was driven by mediators released from virus-infected AECs destroying the function of sodium and chloride transporters along the epithelium rather than its viral infectivity and cytopathic effect [14,21]. We therefore suggest that the relative AFC index is an additional measurement of the potential severity of human disease that may arise from an animal virus infection if it were to cause zoonotic or pandemic disease.

In the recent outbreak of COVID-19, the fast spread globally has created many public health concerns since no prior surveillance or risk assessment has detected a similar isolate. We used *ex vivo* human airway explants model to study the replication, transmissibility and pathogenesis of SARS-CoV-2 [35]. With the quantitative screening platform proposed here, we can further study the AFC using the *in vitro* lung injury model to deduce the potential disease severity of different SARS-CoV-2 variants, in reference to the pandemic H1N1 and HPAI H5N1 viruses.

It is crucial to interpret comparisons of AFC impairment between these viruses carefully, as different MOI values were used in the models. Varying infection doses can influence the extent of viral replication and host responses. Normalizing for these differences or standardizing infection conditions would enhance the validity of such comparative analyses. Since it is practically more convenient to perform virus infection *in vitro*, we have opted to use this approach. We confirmed that the results are not sensitive to MOI within the range of MOI 0.1–10. In *ex vivo* cultures, we used the approach of the two reference viruses, pandemic 2009 H1N1 and HPAI H5N1 483/97, in all experiments for normalization. Seasonal influenza A (H1N1, H3N2) viruses and influenza B viruses affect AFC similarly as the pandemic H1N1 (Figs 3a and S2D), whereas all HPAI H5N1 (except SZ/1) and H7N9 viruses had severe AFC impairment. These results have excellent concordance with clinically observed severity of disease [12,22]. Furthermore, LPAI H5N3 and H7N1 viruses from wild aquatic birds which are not known to cause zoonotic disease, and H9N2 viruses which caused mild illness in humans [36], had relative AFC >50. Interestingly, HPAI H5N8 virus also had high relative AFC. This virus has not caused zoonotic disease although has the H5 HA derived from the A/goose/Guangdong/1/96-like virus lineage that cause severe human disease [24]. What was more surprising was that the two HPAI H5N6 viruses had moderate relative AFC ~50%. These viruses have the H5 HA from clade 2.3.4.4, yet clinically H5N6 viruses do cause severe human disease [37] but infection of children appears to be mild [38].

Regarding coronaviruses, including seasonal strains such as OC43, 229E, NL63 and HKU1 could provide valuable baseline data due to their typically mild pathogenicity. Incorporating them into comparative analyses would help delineate the spectrum of AFC impairment associated with coronavirus infections, highlighting the increased severity seen with emerging strains like SARS-CoV-2.

One apparent limitation of *ex vivo* cultures of human airways and *in vitro* cell model is donor-to-donor variation. However, these physiologically relevant experimental models can mitigate the need for extensive human volunteer participation by providing accessible and ethically permissible platforms to study viral infection and host responses. This approach enables researchers to perform a broader range of experiments, including screening multiple virus subtypes and assessing disease mechanisms, without the ethical and logistical challenges associated with recruiting and sampling human volunteers. Consequently, these models help reduce reliance on human samples while still generating relevant biological insights. A wider range of virus subtypes needs to be screened to suggest a more precise correlation of viral replication versus disease severity.

In summary, we provided advanced methods to improve pandemic risk assessment of animal influenza viruses that can be used to advance current risk assessment algorithms.

## Supplementary material

10.1099/jgv.0.002281 Supplementary Material 1.

## Abbreviations

AEC: alveolar epithelial cell
AFC: alveolar fluid clearance
ALI: acute lung injury
APC: antigen-positive cell
AUC: area under the curve
HPAI: highly pathogenic avian influenza
IHC: immunohistochemistry
IRAT: Influenza Risk Assessment Tool
LPAI: low-pathogenic avian influenza
MOI: multiplicity of infection
TIPRA: Tool for Influenza Pandemic Risk Assessment
