# Supplementary material for: Assessment of influenza virus and coronavirus tropism, replication competence and disease severity in ex vivo and in vitro cultures of the human respiratory tract
Source: J Gen Virol. 2026 Jul 3;107(7):002281. doi: 10.1099/jgv.0.002281 (PMC13331291; doi:10.1099/jgv.0.002281)
Supplement: Supplementary Material 1. [file jgv-107-02281-s001.pdf]

Supplementary Material

Assessment of influenza virus and coronavirus tropism, replication competence and disease severity in ex vivo and in vitro cultures of the human respiratory tract

Denise I.T. Kuok[1], Angel P.Y. Ma[1,2], Rachel H.H. Ching[1,2], Ka Chun Ng[1], Jae W. Lee[3], Michael A. Matthay[4], Yi Guan[1], John M. Nicholls[5], Leo L.M. Poon[1,2], J.S. Malik Peiris[1,2], Kenrie P.Y. Hui[1,2], Michael C.W. Chan[1,2#]

[1]School of Public Health, LKS Faculty of Medicine, The University of Hong Kong, Hong Kong SAR, China.

[2]Centre for Immunology and Infection (C2i), Hong Kong Science Park, Hong Kong SAR, China.

[3]Department of Anesthesiology, University of California, Los Angeles, USA.

[4]Department of Medicine and Anesthesiology, University of California San Francisco, San Francisco, USA.

[5]Department of Pathology, School of Clinical Medicine, LKS Faculty of Medicine, Queen Mary Hospital, The University of Hong Kong, Hong Kong SAR, China.

[#]Corresponding Author.

Correspondence:

Name: Prof. Michael C.W. Chan

Email: mchan@hku.hk

Methods

Viral titration by $TCID_{50}$ assay

Confluent 96-well tissue culture plates of MDCK, Vero E6 or MRC-5 cells were prepared one day before. Cells were washed once with PBS and replenished with serum-free Minimum Essential Media (MEM) for MDCK, Dulbecco's Modified Eagle's Medium (DMEM) for Vero E6 and 2% MEM for MRC-5 cells supplemented with 100 units/ml penicillin and 100 μg/ml streptomycin and 2 μg/ml of TPCK (tosylsulfonyl phenylalanylchloromethyl ketone)-treated trypsin. Serial dilutions of virus supernatant, from 0.5 log to 7 log, were performed before adding to cell plates. The plates incubated at 37°C and 5% CO_2 humidified incubator were observed for cytopathic effect daily. The endpoint of viral dilution causing CPE in 50% of inoculated wells was estimated using the Karber method.

Infection of *ex vivo* culture of human bronchus and lung

Human bronchus and lung tissues were sectioned into thin pieces and submerged into 10^6 $TCID_{50}$/mL virus and incubated at 37°C and 5% CO_2 incubator. Tissues were rinsed off unbound viruses with PBS after 1h incubation. Lung and bronchus tissues were placed into 24-well plate with F-12K nutrient mixture (Gibco) containing 100 U/ml penicillin and 100 μg/ml streptomycin. Bronchus were placed on top of surgical sponge to establish air-liquid interface. Infected tissues were placed back to the 37°C incubator. Tissue supernatants were collected at 1, 24 and 48 hpi for viral replication and tissues were fixed with 10% formalin at 24 and 48 hpi for immunohistochemistry.

Area under curve (AUC) analysis of viral replication competence in *ex vivo* culture

Viral replication competence in *ex vivo* cultures of human bronchus and lung was presented using area under curve (AUC). The viral titer at 1, 24 and 48 hpi obtained from $TCID_{50}$ assay was entered into the Prism software. Total virus release from infected tissues and alveolar epithelial cells (on Transwell) was calculated from the trapezoid area under virus replication kinetic curves to the detection limit of $TCID_{50}$ ($10^{1.5}$) between 24 and 48 hpi (1). Infectious viral titers at 1 hpi may reflect inoculum and were therefore not included in the AUC calculation. We have previously shown that residual inoculum present at 1 hpi has lost infectivity by 24 hours because of thermal inactivation. Thus, virus quantitated at 24 and 48 hpi reflects active

replication in the *ex vivo* tissues. The AUC of *ex vivo* lung and bronchus cultures was determined using GraphPad Prism version 5.0 (GraphPad Software, La Jolla, CA, USA). For *ex vivo* bronchus culture, the AUC of the reference strain pandemic H1N1 (A/Hong Kong/415742/2009) was set as 100 and HPAI H5N1 (A/Hong Kong/483/1997) as 0 for each replicate experiment. The calculated AUC of each test virus was normalized to these reference strains using the calculation: $(AUC_{virus} – AUC_{H5N1})/ AUC_{H1N1}$. Relative AUC index was plotted as dot plot with mean±SEM using GraphPad Prism. At least three independent donors of human lung and bronchus tissues were used for each virus.

Immunohistochemical (IHC) staining

Ex vivo human lungs and bronchus tissues were fixed in 10% formalin at 24 hpi (for influenza A) and 48 hpi (for influenza B and coronaviruses) overnight, paraffin-embedded and sliced into multiple sections (2). These sections were incubated with 0.1% $CaCl_2$ in pH 7.8 containing 0.05% Pronase (Roche, Switzerland) at 37°C for 2 min. After, they were blocked with 3% H_2O_2 in TBS for 10 min and avidin/biotin kit (Vector Lab, USA). Sections were incubated with 10% normal rabbit serum and 15µg/mL mouse anti-influenza A nucleoprotein antibody (HB65, EVL Laboratories, Netherlands), mouse anti-influenza B nucleoprotein monoclonal antibody [8.F.183] (Abcam #119967) (3), rabbit polyclonal anti-MERS-CoV nucleoprotein (Abbiotec #9339A, USA) (4) or mouse monoclonal anti-SARS-CoV nucleoprotein antibody (Similar to 42C) (5) (BEI Resources #NR-619, USA) at room temperature for 1h. Biotinylated rabbit anti-mouse (Dako cytomatin, USA) antibody was conjugated for 30min. Sections were developed using NovaRED peroxidase substrate kit (Vector Lab #SK-5100, USA) and mounted with Permount™ (ThermoFisher, USA) on slide.

Human alveolar epithelial cells isolation

Non-malignant lung tissues taken from patients undergone surgery at the Department of Cardiothoracic Surgery, Queen Mary Hospital, Hong Kong (IRB reference no: UW 09-394) were used for primary alveolar epithelial cell isolation described previously(6). In brief, lung tissues were dissected into pieces and washed with Hank's balanced salt solution (BSS) containing 0.7 mM sodium bicarbonate (Gibco) at pH 7.4. The tissues were digested using a combination of 0.5% trypsin (Gibco) and 4 U/ml elastase (Worthington Biochemical Corporation, Lakewood,

NJ, USA) for 40 min at a 37°C shaking water-bath. The digestion was stopped by adding DMEM/F12 medium (Gibco) with 40% FBS with DNase I (350 U/ml) (Sigma). Cell clumps were dispersed by pipetting the cell suspension for 10 min and gone through disposable cell strainers (gauze size of 50 μm) (BD Bioscience). The flow-through was pelleted and resuspended in a 1:1 mixture of DMEM/F12 medium and small airway growth medium (SAGM) (Lonza), 5% FBS and 350 U/ml DNase I, and seeded into tissue culture flasks and put in a 37°C and 5% CO_2 incubator for 90 min. Non-adherent cells were pelleted and resuspended in SAGM supplemented with 1% FBS, 100 U/ml penicillin and 100 μg/ml streptomycin and plated into new tissue culture flasks. Growth medium was changed daily starting from 60 h after plating the cells. When the cell layer approached 75% confluence, the alveolar epithelial cells were trypsinized and seeded as in the transwell culture inserts.

In vitro lung injury model

An *in vitro* human lung injury model using primary human alveolar epithelial cells (AECs) was used for evaluating alveolar fluid clearance (AFC) of influenza viruses was previously described (7) (See supplementary material for AECs isolation). Briefly, AECs were isolated from non-tumor lung tissues and seeded on the apical surface of inserts of a 24-well transwell plate to establish an air-liquid interface prior to infection. AECs were infected with influenza A and B viruses at multiplicity of infection (MOI) of 0.1 and coronaviruses at MOI 1 for 1 hour at 37°C and 5% CO_2. Then, cells were washed with PBS and replenished with supplemented small airway growth medium (SAGM™, Lonza, USA) containing 12.5ug FITC-labeled dextran (Sigma). Mock-infected AECs were used as negative control. Net AFC was determined by change in fluorescent intensity of dextran in AECs over a 24h-infection period (7). It was calculated as [1 – (initial fluorescence reading/ final fluorescence reading)] × 200μL /0.33 cm^2 /24h. Viral titers of culture supernatant on the apical surface of AECs was measured at 1, 24 and 48 hpi by $TCID_{50}$ assay and AUC was calculated and plotted using Prism. The net AFC values of pandemic H1N1 (415742/09) were set as 100 and HPAI H5N1 as 0 for each experimental replicate. Then, the net AFC of each virus is normalized to these reference strains: $(AFC_{virus} – AFC_{H5N1})/ AFC_{H1N1}$, and relative AFC was plotted in dot plot with mean±SEM using GraphPad Prism. At least three donors of AECs were used for all experiments.

References

1. Gonzalez-Parra G, Rodriguez T, Dobrovolny HM. A comparison of methods for extracting influenza viral titer characteristics. J Virol Methods. **2016**;231:14-24.

2. Hui KP, Chan LL, Kuok DI, Mok CK, Yang ZF, Li RF, et al. Tropism and innate host responses of influenza A/H5N6 virus: an analysis of ex vivo and in vitro cultures of the human respiratory tract. Eur Respir J. **2017**;8(49):3.

3. Bui CHT, Chan RWY, Ng MMT, Cheung MC, Ng KC, Chan MPK, et al. Tropism of influenza B viruses in human respiratory tract explants and airway organoids. Eur Respir J. **2019**;54(2).

4. Chan RW, Hemida MG, Kayali G, Chu DK, Poon LL, Alnaeem A, et al. Tropism and replication of Middle East respiratory syndrome coronavirus from dromedary camels in the human respiratory tract: an in-vitro and ex-vivo study. The Lancet Respiratory medicine. **2014**;2(10):813-22.

5. Nicholls JM, Poon LL, Lee KC, Ng WF, Lai ST, Leung CY, et al. Lung pathology of fatal severe acute respiratory syndrome. Lancet. **2003**;361(9371):1773-8.

6. Chan MC, Chan RW, Chan LL, Mok CK, Hui KP, Fong JH, et al. Tropism and innate host responses of a novel avian influenza A H7N9 virus: an analysis of ex-vivo and in-vitro cultures of the human respiratory tract. The lancet Respiratory medicine. **2013**;1(7):534-42.

7. Chan MC, Kuok DI, Leung CY, Hui KP, Valkenburg SA, Lau EH, et al. Human mesenchymal stromal cells reduce influenza A H5N1-associated acute lung injury in vitro and in vivo. Proc Natl Acad Sci USA. **2016**;113(13):3621-6.

Table S1. List of influenza viruses and coronaviruses evaluated for tissue tropism in *ex vivo* explant infection and disease severity in the *in vitro* human lung injury models. The subtypes, lineages and strains of influenza A, B and coronaviruses with their respective abbreviation were listed. *Viruses isolated from avian surveillance at the Hong Kong Mai Po Natural Reserve.

Virus Strain	Abbreviation	Subtype	Virus isolation origin
**Influenza A**			
A/Hong Kong/54/1998	H1N1 (54/98)	H1N1	Human
A/Oklahoma/447/2008	H1N1 (447/08)	H1N1	Human
A/Hong Kong/415742/2009	H1N1pdm (415742/09)	H1N1pdm	Human
A/Hong Kong/1174/1999	H3N2 (1174/99)	H3N2	Human
A/Oklahoma/1992/2005	H3N2 (1992/05)	H3N2	Human
A/Hong Kong/483/1997	H5N1 (483/97)	H5N1	Human
A/Vietnam/1203/2004	H5N1 (1203/04)	H5N1	Human
A/Shenzhen/1/2012	H5N1 (SZ1/12)	H5N1	Human
A/Hong Kong/MPQ1017/2015*	H5N3 (MPQ1017/15)	H5N3	Wild bird/Avian
A/Guangzhou/39715/2014	H5N6 (39715/14)	H5N6	Human
A/Oriental magpie robin/Hong Kong/6154/2015	H5N6 (6154/15)	H5N6	Wild bird/Avian
A/Northern pintail/Hong Kong/MP5883/2004	H5N8 (MP5583/04)	H5N8	Duck/Avian
A/Hong Kong/MPQ1219/2015*	H7N1 (MPQ1219/15)	H7N1	Wild bird/Avian
A/Shanghai/1/2013	H7N9 (Sh1/13)	H7N9	Human
A/Shanghai/2/2013	H7N9 (Sh2/13)	H7N9	Human
A/Anhui/1/2013	H7N9 (AH1/13)	H7N9	Human
A/Qingyuan/GIRD1/2017	H7N9 (QY/17)	H7N9	Human
A/Quail/Hong Kong/G1/1997	H9N2 (G1/97)	H9N2	Quail/Avian
A/Duck/Hong Kong/Y280/1997	H9N2 (Y280/97)	H9N2	Duck/Avian
**Influenza B**		**Lineage**	
B/Hong Kong/407373/2011	B (407373/11)	Victoria	Human
B/Hong Kong/448799/2012	B (448799/12)	Yamagata	Human
**Coronavirus**		**Strain**	
HCoV-EMC/2012	MERS-CoV	MERS-CoV	Human
SARS-CoV	SARS-CoV	HK39849	Human
SARS-CoV-2	SARS-CoV-2	A	Human

Table S2. Immunohistochemical scoring of ex vivo infected human lung and bronchus tissues. The extent of viral infectivity was denoted by (sparse) ≤ 10 %, (+) 11– 40 %, (++) 41 – 70 %, and (+++) ≥ 71% of the total epithelial cells; (-) denotes no positively stained epithelial cells were located.

Lung	Virus Strain	Bronchus
++	H1N1 (54/98)	+++
+	H1N1 (447/08)	++
+++	H1N1 (415742/09)	++
-	H3N2 (1174/99)	sparse
sparse	H3N2 (1992/05)	sparse
+++	H5N1 (483/97)	sparse
++	H5N1 (1203/04)	sparse
++	H5N1 (SZ1/12)	sparse
++	H5N3 (MPQ1017/15)	-
++	H5N6 (39715/14)	++
++	H5N6 (6154/15)	++
++	H5N8 (MP5883/04)	sparse
+	H7N1 (MPQ1219/15)	sparse
+++	H7N9 (Sh1/13)	++
+++	H7N9 (Sh2/13)	++
+	H7N9 (AH1/13)	++
+++	H7N9 (QY/17)	sparse
+++	H9N2 (G1/97)	+++
+++	H9N2 (Y280/97)	-
++	B (407373/11)	+++
++	B (448799/12)	+++
++	MERS-CoV	+
sparse	SARS-CoV	-
+	SARS-CoV-2	++

A

Viruses	H1N1 (54/98)	H1N1 (447/08)	H1N1 (415742/09)	H3N2 (1174/99)	H3N2 (1992/05)	H5N1 (483/97)	H5N1 (1203/04)	H5N1 (SZ1/12)	H5N3 (MPQ1017/15)	H5N6 (39715/14)	H5N6 (6154/15)	H5N8 (MP5883/04)	H7N1 (MPQ1219/15)	H7N9 (Sh1/13)	H7N9 (Sh2/13)	H7N9 (AH1/13)	H7N9 (QY/17)	H9N2 (G1/97)	H9N2 (Y280/97)	B (407373/11)	B (448799/12)	MERS-CoV	SARS-CoV
H1N1 (54/98)																							
H1N1 (447/08)	ns																						
H1N1 (415742/09)	***	**																					
H3N2 (1174/99)	ns	ns	**																				
H3N2 (1992/05)	ns	ns	***	ns																			
H5N1 (483/97)	**	***	***	***	**																		
H5N1 (1203/04)	***	***	***	***	***	ns																	
H5N1 (SZ1/12)	***	***	***	***	***	ns	ns																
H5N3 (MPQ1017/15)	**	***	***	***	**	ns	ns	ns															
H5N6 (39715/14)	ns	ns	**	ns	ns	***	***	***	**														
H5N6 (6154/15)	**	***	***	***	**	ns	ns	ns	ns	***													
H5N8 (MP5883/04)	ns	ns	***	ns	ns	ns	**	*	ns	ns	ns												
H7N1 (MPQ1219/15)	***	***	***	***	***	ns	ns	ns	ns	***	ns	ns											
H7N9 (Sh1/13)	ns	ns	***	ns	ns	ns	***	**	ns	ns	ns	ns	ns										
H7N9 (Sh2/13)	ns	ns	***	ns	ns	*	***	***	ns	ns	*	ns	*	ns									
H7N9 (AH1/13)	ns	ns	***	ns	ns	ns	**	**	ns	ns	ns	ns	ns	ns	ns								
H7N9 (QY/17)	ns	ns	***	ns	ns	ns	**	*	ns	ns	ns	ns	ns	ns	ns	ns							
H9N2 (G1/97)	ns	ns	***	ns	ns	*	***	***	ns	ns	*	ns	**	ns	ns	ns	ns						
H9N2 (Y280/97)	ns	ns	***	ns	ns	ns	***	**	ns	ns	ns	ns	ns	ns	ns	ns	ns	ns					
B (407373/11)	ns	ns	***	ns	ns	ns	***	***	ns	ns	ns	ns	*	ns	ns	ns	ns	ns	ns				
B (448799/12)	ns	ns	*	ns	ns	***	***	***	***	ns	***	ns	***	ns	ns	ns	ns	ns	ns	ns			
MERS-CoV	ns	ns	ns	ns	ns	***	***	***	***	ns	***	**	***	**	*	**	***	**	**	*	ns		
SARS-CoV	***	***	***	***	***	ns	ns	ns	ns	***	ns	ns	ns	**	***	*	*	***	**	***	***	***	
SARS-CoV-2	ns	ns	***	ns	ns	***	***	***	**	ns	***	ns	***	ns	ns	ns	ns	ns	ns	ns	ns	ns	**

B

Viruses	H1N1 (54/98)	H1N1 (447/08)	H1N1 (415742/09)	H3N2 (1174/99)	H3N2 (1992/05)	H5N1 (483/97)	H5N1 (1203/04)	H5N1 (SZ1/12)	H5N3 (MPQ1017/15)	H5N6 (39715/14)	H5N6 (6154/15)	H5N8 (MP5883/04)	H7N1 (MPQ1219/15)	H7N9 (Sh1/13)	H7N9 (Sh2/13)	H7N9 (AH1/13)	H7N9 (QY/17)	H9N2 (G1/97)	H9N2 (Y280/97)	B (407373/11)	B (448799/12)	MERS-CoV	SARS-CoV
H1N1 (54/98)																							
H1N1 (447/08)	ns																						
H1N1 (415742/09)	ns	ns																					
H3N2 (1174/99)	ns	ns	ns																				
H3N2 (1992/05)	ns	ns	ns	ns																			
H5N1 (483/97)	ns	ns	ns	ns	ns																		
H5N1 (1203/04)	ns	ns	ns	ns	ns	ns																	
H5N1 (SZ1/12)	ns	ns	ns	ns	ns	ns	ns																
H5N3 (MPQ1017/15)	***	***	***	ns	*	**	*	ns															
H5N6 (39715/14)	ns	ns	ns	ns	ns	ns	ns	ns	ns														
H5N6 (6154/15)	***	***	***	**	*	***	**	**	ns	ns													
H5N8 (MP5883/04)	*	**	**	ns	ns	ns	ns	ns	ns	ns	ns												
H7N1 (MPQ1219/15)	***	***	***	**	***	***	***	**	ns	ns	ns	ns											
H7N9 (Sh1/13)	ns	ns	ns	ns	ns	ns	ns	ns	ns	ns	ns	ns	ns										
H7N9 (Sh2/13)	ns	ns	ns	ns	ns	ns	ns	ns	ns	ns	ns	ns	ns	ns									
H7N9 (AH1/13)	ns	ns	ns	ns	ns	ns	ns	ns	ns	ns	ns	ns	ns	ns	ns								
H7N9 (QY/17)	ns	ns	ns	ns	ns	ns	ns	ns	ns	ns	ns	ns	ns	ns	ns	ns							
H9N2 (G1/97)	ns	ns	ns	ns	ns	ns	ns	ns	ns	ns	ns	ns	ns	ns	ns	ns	ns						
H9N2 (Y280/97)	ns	ns	ns	ns	ns	ns	ns	ns	ns	ns	ns	ns	*	ns	ns	ns	ns	ns					
B (407373/11)	***	***	***	*	*	**	*	ns	ns	ns	ns	ns	ns	ns	ns	ns	ns	ns	ns				
B (448799/12)	***	***	**	ns	ns	**	*	ns	ns	ns	ns	ns	ns	ns	ns	ns	ns	ns	ns	ns			
MERS-CoV	ns	ns	ns	ns	ns	ns	ns	ns	ns	ns	ns	ns	*	ns	ns	ns	ns	ns	ns	ns	ns		
SARS-CoV	***	***	***	ns	*	**	*	ns	ns	ns	ns	ns	ns	ns	ns	ns	ns	ns	ns	ns	ns	ns	
SARS-CoV-2	***	***	***	***	***	***	***	***	ns	**	ns	ns	ns	ns	ns	ns	*	ns	**	ns	ns	**	ns

C

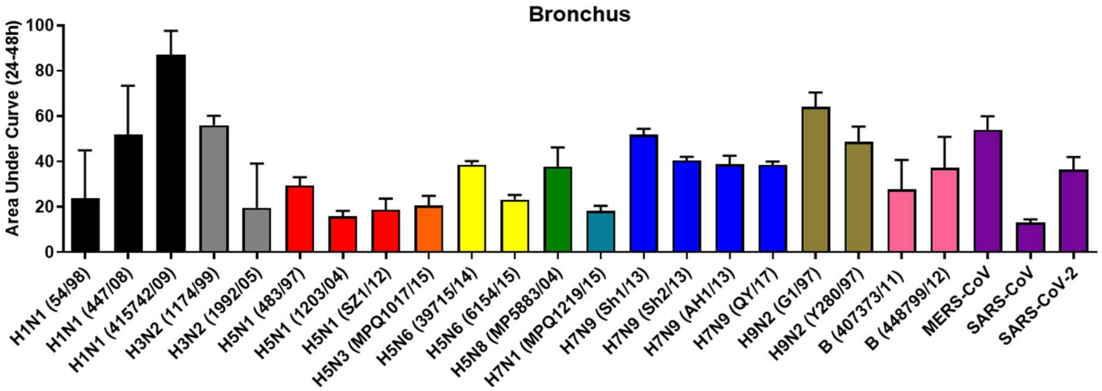

Figure S1. Statistical comparison of Area Under Curve (AUC) of ex vivo infected human airways by influenza viruses and coronaviruses. *Ex vivo* infection of A) human bronchus and B) lung tissues used 10^6 $TCID_{50}$/mL infectious dose at 37°C and viral titer in the culture supernatant at 1, 24 and 48 hpi was measured using the $TCID_{50}$ assay. AUC of replication kinetic in lungs was calculated by Prism, with the AUC replication kinetics in bronchus normalized to those of two reference strains (pandemic H1N1 and H5N1) as relative AUC. Statistical comparison of relative AUC between different viruses was done by one-way ANOVA with Bonferroni's post-test in Prism. C) Viral titers in the culture supernatant of infected tissues were measured at 1, 24 and 48 hpi using TCID50 assay. Area under curve (AUC) was calculated from replication kinetic between 24-48 hpi ($n \geq 3$). *$p<0.05$, **$p<0.01$, ***$p<0.001$.

A

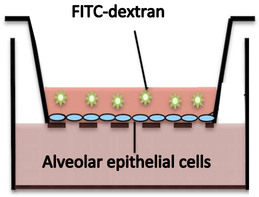

B

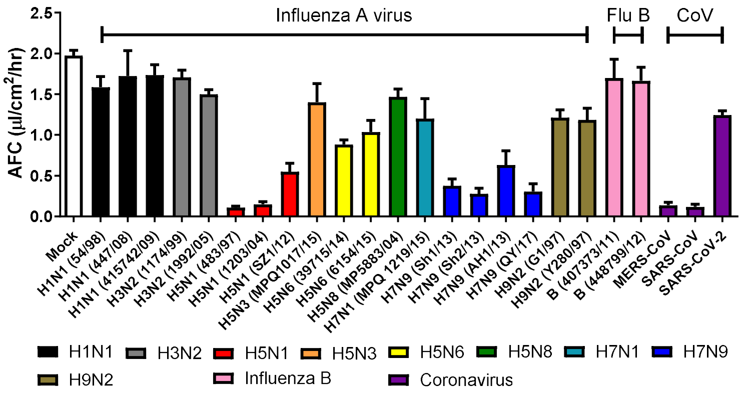

C

Viruses	Mock	H1N1 (54/98)	H1N1 (447/08)	H1N1 (415742/09)	H3N2 (1174/99)	H3N2 (1992/05)	H5N1 (483/97)	H5N1 (1203/04)	H5N1 (SZ1/12)	H5N3 (MPQ1017/15)	H5N6 (39715/14)	H5N6 (6154/15)	H5N8 (MP5883/04)	H7N1 (MPQ1219/15)	H7N9 (Sh1/13)	H7N9 (Sh2/13)	H7N9 (AH1/13)	H7N9 (QY/17)	H9N2 (G1/97)	H9N2 (Y280/97)	B (407373/11)	B (448799/12)	MERS-CoV	SARS-CoV
H1N1 (54/98)	ns																							
H1N1 (447/08)	ns	ns																						
H1N1 (415742/09)	ns	ns	ns																					
H3N2 (1174/99)	ns	ns	ns	ns																				
H3N2 (1992/05)	ns	ns	ns	ns	ns																			
H5N1 (483/97)	***	***	***	***	***	***																		
H5N1 (1203/04)	***	***	***	***	***	***	ns																	
H5N1 (SZ1/12)	***	***	***	***	***	***	ns	ns																
H5N3 (MPQ1017/15)	ns	ns	ns	ns	ns	ns	***	***	***															
H5N6 (39715/14)	***	***	***	***	***	***	***	**	ns	***														
H5N6 (6154/15)	***	***	ns	***	***	ns	***	***	*	ns	ns													
H5N8 (MP5883/04)	***	ns	ns	***	ns	ns	***	***	**	ns	ns	ns												
H7N1 (MPQ1219/15)	*	ns	ns	ns	ns	ns	***	***	***	ns	***	ns	ns											
H7N9 (Sh1/13)	***	***	***	***	***	***	ns	ns	ns	***	ns	**	***	***										
H7N9 (Sh2/13)	***	***	***	***	***	***	ns	ns	ns	***	ns	***	***	***	ns									
H7N9 (AH1/13)	***	***	***	***	***	***	ns	ns	ns	***	ns	ns	*	***	ns	ns								
H7N9 (QY/17)	***	***	***	***	***	***	ns	ns	ns	***	*	***	***	***	ns	ns	ns							
H9N2 (G1/97)	***	ns	ns	**	ns	ns	***	***	***	ns	ns	ns	ns	ns	***	***	*	***						
H9N2 (Y280/97)	***	ns	ns	**	ns	ns	***	***	***	ns	**	ns	ns	ns	***	***	***	***	ns					
B (407373/11)	ns	ns	ns	ns	ns	ns	***	***	***	ns	***	***	*	ns	***	***	***	***	ns	ns				
B (448799/12)	ns	ns	ns	ns	ns	ns	***	***	***	ns	***	ns	ns	ns	***	***	***	***	ns	ns	ns			
MERS-CoV	***	***	***	***	***	***	ns	ns	ns	***	ns	***	***	***	ns	ns	ns	ns	***	***	***	***		
SARS-CoV	***	***	***	***	***	***	ns	ns	ns	***	*	***	***	***	ns	ns	ns	ns	***	***	***	***	ns	
SARS-CoV-2	***	ns	ns	***	ns	ns	***	***	***	ns	**	ns	ns	ns	***	***	***	***	ns	ns	*	ns	***	***

D

Viruses	H1N1 (54/98)	H1N1 (447/08)	H1N1 (415742/09)	H3N2 (1174/99)	H3N2 (1992/05)	H5N1 (483/97)	H5N1 (1203/04)	H5N1 (SZ1/12)	H5N3 (MPQ1017/15)	H5N6 (39715/14)	H5N6 (6154/15)	H5N8 (MP5883/04)	H7N1 (MPQ1219/15)	H7N9 (Sh1/13)	H7N9 (Sh2/13)	H7N9 (AH1/13)	H7N9 (QY/17)	H9N2 (G1/97)	H9N2 (Y280/97)	B (407373/11)	B (448799/12)	MERS-CoV	SARS-CoV
H1N1 (54/98)																							
H1N1 (447/08)	*																						
H1N1 (415742/09)	ns	ns																					
H3N2 (1174/99)	ns	ns	ns																				
H3N2 (1992/05)	***	ns	ns	ns																			
H5N1 (483/97)	***	ns	ns	**	ns																		
H5N1 (1203/04)	***	ns	**	***	ns	ns																	
H5N1 (SZ1/12)	ns	***	***	***	***	***	***																
H5N3 (MPQ1017/15)	ns	***	*	ns	***	***	***	ns															
H5N6 (39715/14)	***	***	***	***	***	**	ns	***	***														
H5N6 (6154/15)	***	ns	ns	ns	ns	ns	ns	***	***	***													
H5N8 (MP5883/04)	**	***	***	***	***	***	***	ns	ns	***	***												
H7N1 (MPQ1219/15)	**	***	***	***	***	***	***	ns	ns	***	***	ns											
H7N9 (Sh1/13)	ns	ns	ns	ns	ns	ns	ns	***	**	***	ns	***	***										
H7N9 (Sh2/13)	***	ns	ns	**	ns	ns	ns	***	***	*	ns	***	***	ns									
H7N9 (AH1/13)	**	ns	ns	ns	ns	ns	ns	***	***	***	ns	***	***	ns	ns								
H7N9 (QY/17)	***	ns	ns	ns	ns	ns	ns	***	***	***	ns	***	***	ns	ns	ns							
H9N2 (G1/97)	ns	ns	ns	ns	ns	ns	ns	***	***	***	ns	***	***	ns	ns	ns	ns						
H9N2 (Y280/97)	ns	ns	ns	ns	**	***	***	*	ns	***	**	***	**	ns	***	*	***	ns					
B (407373/11)	ns	ns	ns	ns	*	***	***	**	ns	***	ns	***	***	ns	***	ns	*	ns	ns				
B (448799/12)	ns	ns	ns	ns	ns	*	**	***	ns	***	ns	***	***	ns	*	ns	ns	ns	ns	ns			
MERS-CoV	*	ns	ns	ns	ns	ns	ns	***	***	***	ns	***	***	ns	ns	ns	ns	ns	ns	ns	ns		
SARS-CoV	**	***	***	***	***	***	***	ns	ns	***	***	ns	ns	***	***	***	***	***	***	***	***	***	
SARS-CoV-2	***	***	***	***	***	***	***	*	***	***	***	ns	ns	***	***	***	***	***	***	***	***	***	ns

Figure S2. Alveolar fluid clearance of influenza viruses and coronaviruses in vitro. A) Transwell lung injury model. B) Alveolar fluid clearance (AFC) of different viruses at 24 hpi ($n \geq 3$, mean±SEM). Statistical comparison of C) relative AFC and D) AUC of different viruses by one-way ANOVA with Bonferroni's post-test. *$p<0.05$, **$p<0.01$, ***$p<0.001$.

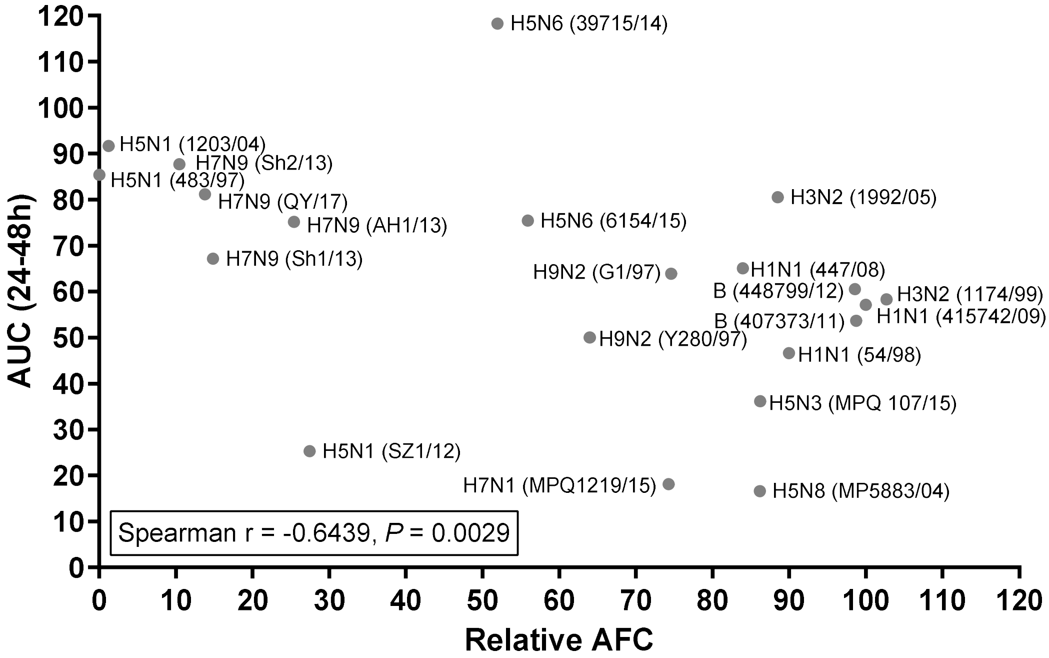

Figure S3. Correlation between alveolar epithelial cell viral replication and relative alveolar fluid clearance (AFC) in the in vitro lung injury model. Each dot represents the area under curve (AUC) at 24-48 hpi of viral replication kinetic and relative AFC (at 24 hpi) of different influenza virus strain respectively. r and P values are for Spearman rank correlation test calculated by Prism, **p<0.01.

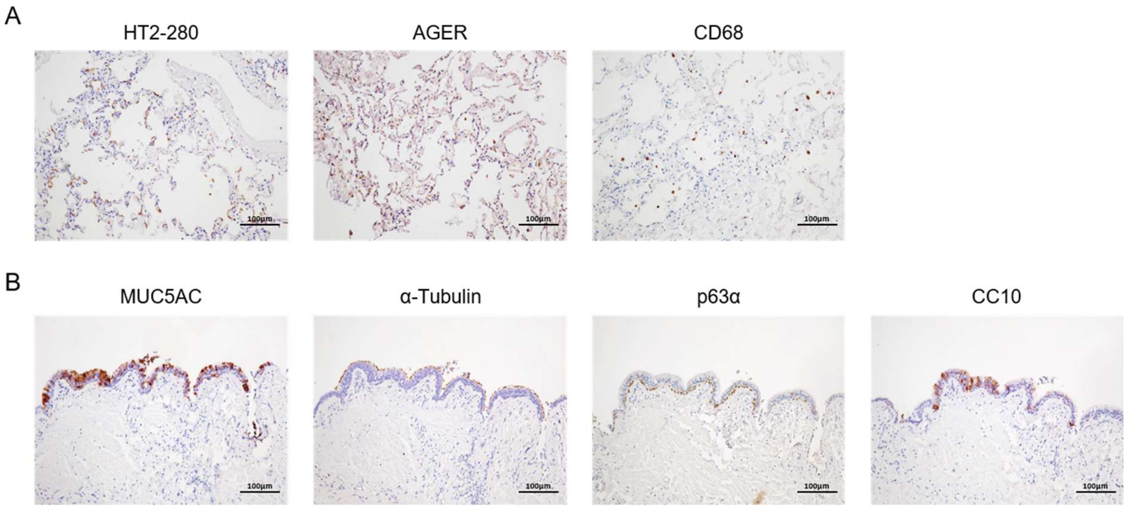

Figure S4. Characterization of the lung and bronchus ex vivo models by IHC. Cell marker staining specifically for the cell types found in the (A) lungs and (B) bronchus were performed. Positively stained cells are in red or brown. HT2-280: alveolar type 2 cells, AGER: alveolar type 1 cells, CD68: macrophages, Muc5Ac: goblet cells, α-tubulin: ciliated cells, p63α: basal cells, and CC10: club cells. Scale bar = 100 µm.
